# Characteristics and risk assessment of occupational exposure to ultrafine particles generated from cooking in the Chinese restaurant

**DOI:** 10.1038/s41598-021-95038-y

**Published:** 2021-08-02

**Authors:** Xiangjing Gao, Meibian Zhang, Hua Zou, Zanrong Zhou, Weiming Yuan, Changjian Quan, Yiyao Cao

**Affiliations:** Department of Occupational Health and Radiation Protection, Zhejiang Provincial Center for Disease Control and Prevention, Hangzhou, 310051 Zhejiang China

**Keywords:** Environmental sciences, Health occupations

## Abstract

Ultrafine particles have been increasingly linked to adverse health effects in restaurant workers. This study aimed to clarify the exposure characteristics and risks of ultrafine particles during the cooking process, and to provide a reasonable standard for protecting the workers in the Chinese restaurant. The temporal variations in particle concentrations (number concentration (NC), mass concentration (MC), surface area concentration (SAC), and personal NC), and size distributions by number were measured by real-time system. The hazard, exposure, and risk levels of ultrafine particles were analyzed using the control banding tools. The NC, MC, and SAC increased during the cooking period and decreased gradually to background levels post-operation. The concentration ratios of MC, total NC, SAC, and personal NC ranged from 3.82 to 9.35. The ultrafine particles were mainly gathered at 10.4 and 100 nm during cooking. The exposure, hazard and risk levels of the ultrafine particles were high. These findings indicated that the workers during cooking were at high risk due to exposure to high levels of ultrafine particles associated with working activity and with a bimodal size distribution. The existing control strategies, including engineering control, management control, and personal protection equipment need to be improved to reduce the risk.

## Introduction

Cooking is an essential daily activity in households and also some commercial spaces. However, the cooking process can generate a large amount of particulate matter (PM) of different sizes, ranging from ultrafine (< 0.1 μm) to accumulation (0.1–2.0 μm) and coarse modes (> 2.0 μm)^1^. Large amounts of harmful PM that contain polycyclic aromatic hydrocarbons (PAHs), volatile organic compounds, and carbonyl compounds were generated during the cooking process^2^. Chowdhury et al. reported that 43.2% of the nanoparticles and 52.5% of microparticle exposure were from the cooking process^3^. Many studies have shown that cooking could be one of the major indoor sources of PM in total particle exposure^4–7^ and contribute to approximately 30% of the indoor particle concentration in the size range of 0.5–5 μm^8^.

It has been found that particles at the nanoscale level cause more adverse health effects than larger particles with the same components^9^. In vitro and in vivo experiments have reported that the particles generated from cooking could deposit in the lungs, leading to adverse health effects on both the respiratory and cardiovascular systems, including decreased lung function, asthma, myocardial infarction, all-cause mortality, and cancer^10–13^. Epidemiological investigations have also demonstrated a significant link between negative health effects and exposure to PM from cooking^7,14^.

Exposure characterization for PM from cooking has been a topic of academic interest in recent years. Many studies have investigated indoor particle dispersion and spatial exposure due to cooking activities. One study on indoor air pollution from gas cooking at home based on 24-h monitoring reported that ultrafine particle NC increased to 1.4 × 10^6^ particles/cm^3^ and the average MC of PM_10_ increased to 80 μg/m^3^ during cooking^15^. The 24-h time-resolved measurements of PM_2.5_ found that the median PM_2.5_ concentration was 79 μg/m^3^, and the NC of particle was 8.5 × 10^4^ particles/cm^3^^16^. The emissions and indoor concentrations of PM in NC and MC under different cooking conditions were reviewed in a previous study^17^. This review found that cooking can generate a notable volume of particles with a respirable size range, and the chemical compounds included alkanes, fatty acids, dicarboxylic acids, lactones, PAHs, alkenones, and sterols. Previous studies of exposure assessment mostly focused on the characteristics of PM associated with residential cooking activities at home. Few studies have reported the characteristics of ultrafine particles exposed to kitchen workers in Chinese restaurants. Due to the rapid development of the catering industry and the proximity these workers have to PM, a large number of occupational workers in the kitchen may be at high risk of exposure to ultrafine particles, which should be a cause for concern.

Exposure characterization is the basis for exposure assessment and risk assessment. Along with the exposure characterization, a carcinogenic risk assessment of the chemical compositions of the ultrafine particles involved in cooking was conducted. A health risk assessment of the volatile organic compounds (VOCs) from cooking in restaurants has been reported^18^. A chemical characterization and cancer risk assessment for airborne carbonyls, particulate-bound PAHs, and heavy metals was conducted under low ventilation conditions in eight categories of commercial restaurants^19^. Previous risk assessment studies have focused on the chemical components involved in cooking. However, the components of the ultrafine particles generated from cooking are complex, and their respective exposure characterization is difficult to obtain. Moreover, most occupational exposure limits for the components of the ultrafine particles from cooking are lacking. In order to manage the risks of the ultrafine particles from cooking, a feasible and easy risk assessment strategy that can classify the substance into risk categories with recommended control measures based on limited physicochemical and task/scenario information should be introduced. The control banding (CB) tools evaluate the hazard risk and exposure risk by setting different parameters, and then obtain risk levels and protective measures based on a tiered approach. The CB tools, which offer simplified guidance for managing the risks from exposure to substances without toxicological and/or detailed exposure information, are applicable methods and have been used for risk assessments of different ultrafine particle exposure levels in various work environments^20–23^. In previous studies, CB tools were compared qualitatively and quantitatively under experimental or real nanomaterial exposure scenarios, and different tools to estimate hazard and exposure bands can result in different outcomes and preventive recommendations^22–25^.

The processes used in Chinese restaurants generally operate under rapid high-heat conditions, such as frying and roasting, which are different from Western restaurants, may contribute significantly to the emissions of ultrafine particles. Knowledge of the exposure characteristics and risk assessments of ultrafine particles for workers in Chinese restaurants is limited. In order to bridge the above research gaps, it is necessary to understand the exposure characteristics and risks of ultrafine particles generated during cooking comprehensively, and provide a basis for developing a reasonable control strategy to reduce the health risks for workers in Chinese restaurants.

In order to understand the exposure characteristics and the risks of ultrafine particles during cooking, and provide a basis for developing a reasonable control measures to reduce the health risks for workers, the following three aspects were investigated in this study: (1) the temporal variation in total number concentrations of particles (namely, total NC, total respirable MC, personal NC, and SAC) and size distributions by number; (2) the risk levels of workers exposed to ultrafine particles; (3) the control measures to be improved for reducing the health risks of workers.

## Materials and methods

### Description of workplace

One commercial Chinese restaurant in the Zhejiang Province of East China was selected for a field investigation. The major foods of the restaurant that were served covered meats, vegetables, fishes, shrimp, eggs, and noodles. The main cooking methods included stir-frying, pan-frying, and deep-frying, which were generally operate under rapid high-heat conditions. The fuel used in this restaurant was natural gas. In each shift there were four chefs and five prep cooks wearing working clothes without respiratory protective equipment. Their daily working time is about 4 h. The kitchen door was kept closed at all times. A local exhaust ventilation (LEV) system, which was kept on during cooking, covered all of the stoves in the kitchen. The LEV is a side suction exhaust hood, which is about 0.8 m away from the gas stove.

### Monitoring systems and quality control

Table 1 shows the real-time monitoring system for particles from the cooking process. The total NC was determined using an ultrafine particle counter (Model 3007; TSI, Shoreview, MN). The personal NC and particle size were measured using a Diffusion Size Classifier Miniature (DiSCmini, Testo, Germany), which can measure the number and average size of particles (approximately < 0.7 μm) in the air. The MC was tested using a real-time aerosol monitor (DustTrak 8533, TSI, Shoreview, MN). A surface area monitor (Aero TrakTM 9000, TSI, USA) was used to determine the alveolus deposition model and tracheobronchial (TB) deposition model of SAC. A scanning mobility particle sizer (SMPS, Model 3034, TSI, USA) and optical particle sizer (OPS, Model 3330, TSI, USA) were used jointly to capture the particle size distribution and NC covering the size range from 10.4 nm to 10 μm. The mode size, median size, arithmetic mean size, and geometric mean size were obtained from the data output of the SMPS and OPS. The air velocity was measured by hot-wire anemometer (9515, TSI, USA).

**Table 1:** Monitoring system for measuring the particles from cooking.

Monitoring types	Exposure metrics	Instruments	Particle sizes (nm)	Measuring range	Sampling rate (L/min)	Log interval (min)
Real-time monitoring	Total NC	3007 (TSI, USA)	10–1000	0–100,000 particles/cm^3^ (pt/cm^3^)	0.1	1
	Personal NC	DiSCmini (TESTO, Germany)	< 700	0–5,000,000 pt/cm^3^	1.0	1
	Total respirable MC	Dust Trak 8533 (TSI, USA)	100–1000	0.01–150 mg/m^3^	3	1
	SAC	Aero TrakTM 9000 (TSI, USA)	10–1000	1–10,000 μm^2^/cm^3^	2.5	1
	Size distribution by number	SMPS 3034 (TSI, USA)	10–487	1–2.4 × 10^6^ pt/cm^3^	1.0	3
		OPS 3330 (TSI, USA)	300–10,000	0–3000 pt/cm^3^	1.0	1

All of instruments used were calibrated by its own manufactures (TSI and Testo) once a year. Before measuring, the instruments were set to zero and their inlet filter were cleaned in clean environment. Then, the instruments mode were set as recording mode and set continuous measurement time and data recording interval time.

### Sampling and testing strategies

**Figure 1:**
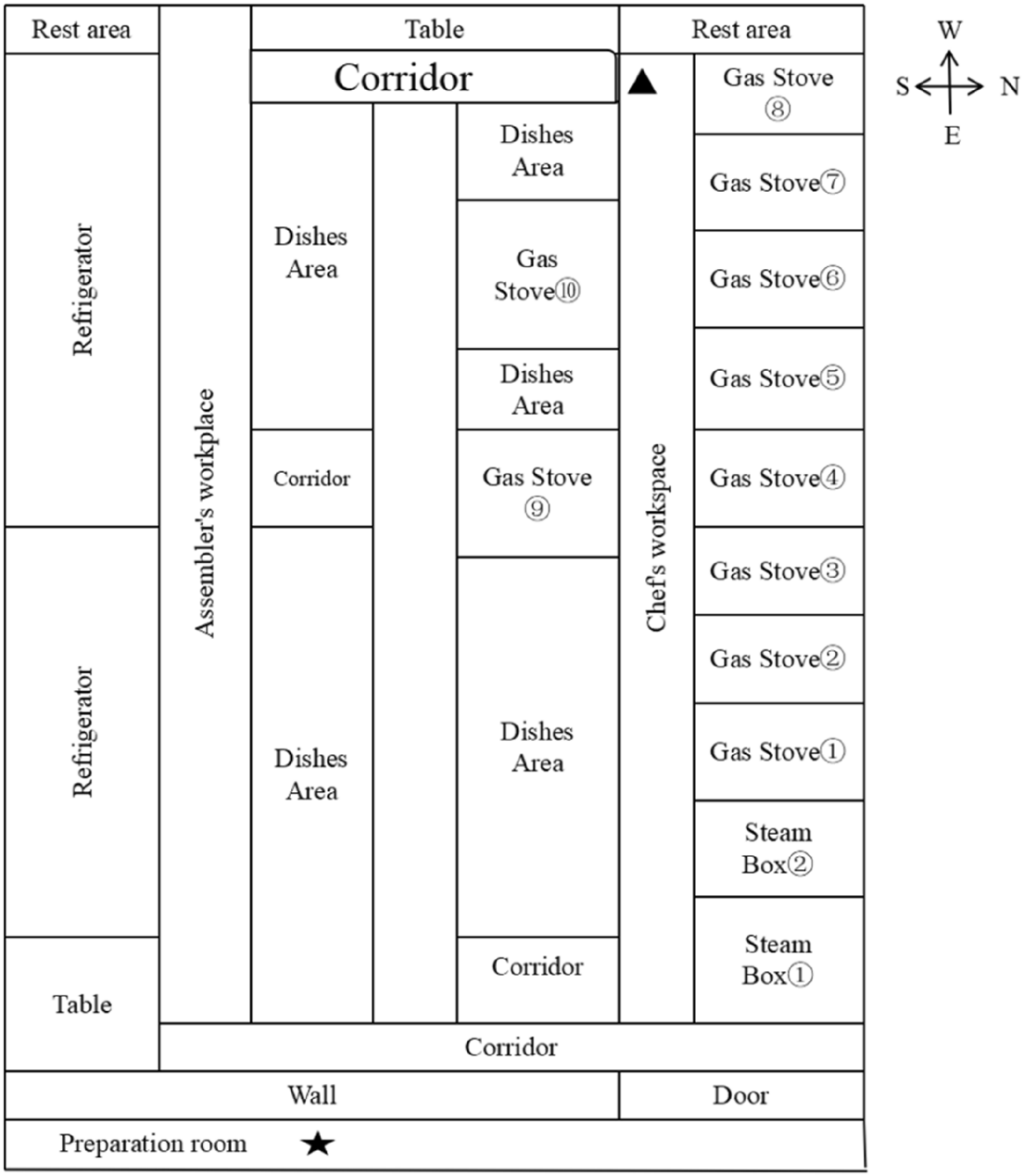
Workshop layout and sampling locations marked with a triangle and an asterisk. Sampling filled star: background; filled triangle: operation location.

The sampling process was performed in October 2019. After the field investigation, concentration screening using the condensation particle counter (CPC) was conducted to identify the potential source of particle emissions, as reported in our previous study^21,22^. The sampling protocol was as follows: (1) Background measurements: background particles from the atmosphere were collected in the preparation room, as shown in Fig. 1 (asterisk location). (2) Area sampling based on activity: the sampling locations were selected near the operation location, considering several factors, such as air movement and currents, work tasks, temperature of the heat source, and whether the location could allow for the placement of large instruments without affecting normal work activities. In this study, all of the stoves were substantial sources of ultrafine particles. However, the corridor that the chefs stand in was too narrow to place the testing instruments in. Hence, the stove numbered 8, which was near the corridor and the rest area, was selected as the sampling location, as shown in Fig. 1 (triangle labeling). Area sampling covered one complete workflow. The instruments’ inlets were positioned 1.3 m above the floor and close to the stove. (iii) Personal sampling: one chef and prep cook station were selected as the personal sampling location, and the sampling period covered one complete workflow. The sampler was fixed approximately 1.3 m above the floor and 30 cm away from the breathing zone of chef or prep cook.

The total concentrations were revised using background concentrations to obtain the concentration ratios (CR) (sampling location vs. background), which reflect the degree of nanoparticles released from the particle generation source. The risk ratio (RR), a relative risk level, which is defined as the ratio between the risk level obtained from the CB tool and the maximum risk level for that tool was introduced to compare risk assessment results across different tools. Similarly, the exposure band ratio is defined as the ratio between the exposure level and the maximum exposure level for the tool, whereas the hazard band ratio is the ratio between the hazard level and the maximum hazard level for the tool. All of the ratios were used to compare the assessment results obtained from different CB tools.

### Methodology for risk assessment

According to previous studies, the Stoffenmanager-Nano and Nanotool, which are appropriate for nanoparticles and have a comprehensive advantage in risk assessment^22,23^, were selected for this study. The CB methodology for CB tools is briefly described as follows: The Stoffenmanager-Nano (http://nano.stoffenmanager.nl/), which follows a stepwise binary decision tree and provides three risk levels^26,27^, was developed by the Organization for Applied Scientific Research based in the Netherlands. The Nanotool (http://www.controlbanding.net/) was developed by Paik and Zalk et al. at the Lawrence Livermore National Laboratory, USA^28^. It assigns the hazard and exposure levels using a point scoring system. The hazard and exposure levels are combined to obtain the risk level in a two-dimensional decision matrix, then is equally divided into four levels^21,28^. Table 2 shows the hazard input data, and Table 3 shows the exposure input data for the CB tools. The hazards were determined from information presented as data on the shape, concentration, surface activity, and toxicity of particles, which includes carcinogenicity, mutagenicity, and reproductive toxicity. The exposure levels were determined by the substance emission potential, activity emission potential, and exposure control, as previously reported.

**Table 2:** Hazard input data of the evaluated materials required by different control banding (CB) tools.

CB tools	Materials information requested	Oil fume
Stoffenmanager-nano	Product appearance	Liquid with medium viscosity (like oil)
	Do you know the exact concentration of the nano component in the product?	No
	Concentration	Main component (50–99%)
	Does the product contain fibers/fiber like particles?	No
	Inhalation hazard	Carcinogenic and mutagenic
	Is the primary particle diameter larger than 50 nm?	No
Nanotool-*parent material*	Current engineering control	Local exhaust ventilation
	Carcinogen	Yes
	Reproductive hazard	No
	Mutagen	Yes
	Dermal hazard	No
	Asthmagen	No
Nanotool-*nanoscale material*	Surface reactivity	Unknown
	Particle shape	Anisotropic
	Particle diameter	11–40 nm
	Solubility	Insoluble
	Carcinogen	Yes
	Reproductive hazard	No
	Mutagen	Yes
	Dermal hazard	No
	Asthmagen	No

**Table 3:** Exposure scenario data input for the particles generated from cooking.

CB tools	Materials information requested	Cooking oil fume
Stoffenmanager Nano	Task characterization	Chemical vapor condensation
	Is the task being carried out in the breathing zone of an employee (distance head-product < 1 m)	Yes
	Is there more than one employee carrying out the same task simultaneously	Yes
	Is the working room being cleaned daily?	Yes
	Are inspections and maintenance of machines/ancillary equipment being done at least monthly to ensure good condition and proper functioning and performance?	No
	Volume of the working room	< 100 m^3^
	Ventilation of the working room	Mechanical and or natural ventilation
	Local control measures	Local exhaust ventilation
	Is the employee situated in a cabin	No
	Is personal protective equipment applied?	No
Nanotool	Chemical vapor condensation	11–100 mg
	Current Engineering Control	Local exhaust ventilation
	Number of Employees with Similar Exposure	6–10
	Frequency of Operation (annual)	Daily
	Operation duration	> 4 h

### Statistical analysis

The differences in the particle concentrations corresponding to different periods was analyzed using the one-way analysis of variance (ANOVA); the LSD comparison method was used when variances were equal, and the Dunnett T3 comparison method was used when the variances were heterogeneous.

### Ethics approval

This study does not involve experimental animals or human participants.

## Results

### Temporal variations in the total particle concentrations

**Figure 2:**
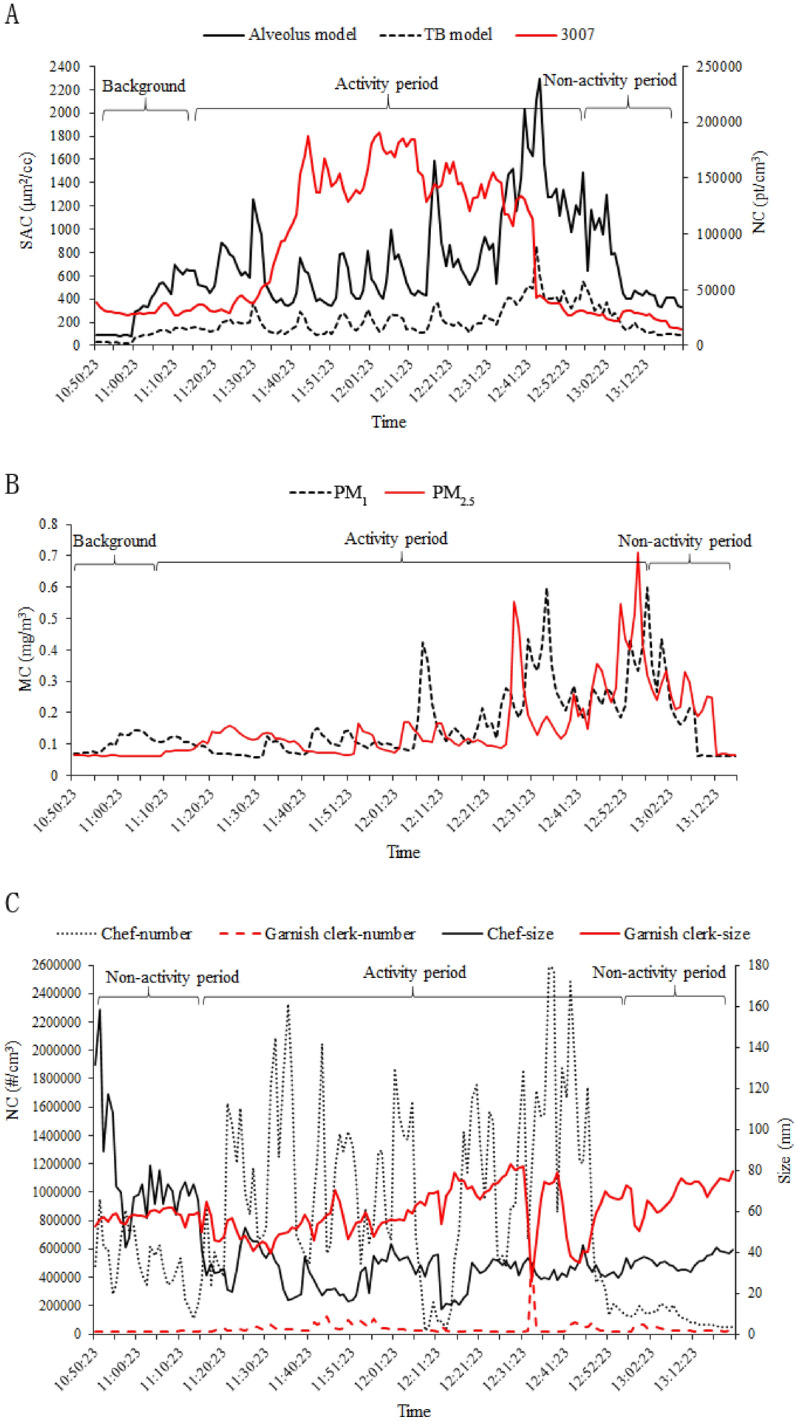
Temporal variations in total particle concentrations associated with working activities. (**A**) Temporal variations in total surface area concentration (SAC) and number concentrations (NC) at background and operation locations. (**B**) Temporal variations in total respirable mass concentration (MC) at the background and operation locations. (**C**) Temporal variations in personal NC and its size during the working and non-activity periods.

The temporal variations in the total particle concentrations are shown in Fig. 2. The total NC during the activity period was higher than that during the non-activity period or background. The highest total NC of the activity period reached 2 × 10^5^ pt/cm^3^ around 30 min after cooking, which was approximately four times higher than that of the non-activity period or background. The highest SACs of the activity period in the alveolus model and TB model were 2400 μm^2^/cm^3^ and 800 μm^2^/cm^3^, respectively, which were approximately five times and three times higher than that of the background (non-activity period), respectively. The MC of PM_2.5_ during the working activity period reached 0.8 mg/cm^3^, approximately ten times higher than that of the non-activity period. The highest NC of the chef reached a peak of 2.6 × 10^6^ pt/cm^3^ at 12:35 during the activity period, which was more than 10 times higher than the non-activity period and the total NC. The NC of the prep cook was lower than 6 × 10^4^ pt/cm^3^, which was approximately 100 times lower than that of the chef. The total NC varied with working activities, which increased after work started and decreased gradually to background level after the activity stopped. The total SAC varied with the activities, but was slightly inconsistent with the time. The total SAC increased before the activity started, and decreased slowly after the operation stopped. The SAC in the alveolus model was higher than that in the TB model, but the variations in the SAC in the alveolus and TB models were similar. The MC of PM_1_ and PM_2.5_ increased near the end of the activity period, and decreased to the background level approximately 30 min after the activity stopped. The total NC and SAC fluctuated greatly during the activity period, while MC performed steadily. Figure 2C shows that the size of the particles exposed to the chef was approximately 40 nm during cooking, while the background particle size was larger than 60 nm. The largest size of the background particles reached 160 nm. The size of the particles exposed to the prep cook remained steady, at around 60 nm.

### Total ultrafine particle concentrations during cooking, the non-activity period, and the background

The total particle concentrations during different periods are listed in Table 4. The NC, PM_1_, PM_2.5_, and SAC during cooking were 14.51 ± 2.50◊10^4^/cm^3^, 0.25 ± 0.11 mg/m^3^, 0.28 ± 0.13 mg/m^3^, 799.20 ± 407.79 μm/cm^3^, and 236.45 ± 129.84 μm/cm^3^, respectively, which were significantly higher than that of the background (*p* < 0.01). The NC, PM_1_, PM_2.5_, and SAC during the non-activity period were 2.82 ± 0.77◊10^4^/cm^3^, 0.07 ± 0.02 mg/m^3^, 0.07 ± 0.02 mg/m^3^, 136.86 ± 47.98 μm/cm^3^, and 45.92 ± 23.64 μm/cm^3^, respectively, which were similar to that of the background. The CR for NC, PM_1_, PM_2.5_, and SAC during cooking ranged from 3.82 to 5.40. The NCs of the chef and prep cook during cooking were 11.53 ± 6.06◊10^5^/cm^3^ and 4.63 ± 0.64◊10^5^/cm^3^, respectively, which were significantly higher than those during the non-activity period.

**Table 4:** Total particle concentrations during cooking, background, and non-activity period.

Metrics	Cooking		Background		Non-activity period	
	Mean ± SD	CR	Mean ± SD	CR	Mean ± SD	CR
NC (10^4^ pt/cm^3^)	14.51 ± 2.50 (n = 65)^a^	5.40	2.69 ± 0.83 (n = 45)	1.00	2.82 ± 0.77 (n = 40)	1.05
PM_1_ (mg/m^3^)	0.25 ± 0.11 (n = 65)^a^	3.85	0.07 ± 0.01 (n = 45)	1.00	0.07 ± 0.02 (n = 40)	1.00
PM_2.5_ (mg/m^3^)	0.28 ± 0.13 (n = 65)^a^	3.93	0.07 ± 0.01 (n = 45)	1.00	0.07 ± 0.02 (n = 40)	1.01
SAC_A model_ (μm^2^/cm^3^)	799.20 ± 407.79 (n = 65)^a^	3.82	106.46 ± 42.28 (n = 45)	1.00	136.86 ± 47.98 (n = 40)	1.28
SAC_TB model_ (μm^2^/cm^3^)	236.45 ± 129.84 (n = 65)^a^	5.15	40.09 ± 28.32 (n = 45)	1.00	45.92 ± 23.64 (n = 40)	1.15
Personal NC-chef (10^5^ pt/cm^3^)	11.53 ± 6.06 (n = 75)^b^	9.35	–	–	1.23 ± 0.81 (n = 58)	1.00
Personal NC-prep cook (10^4^ pt/cm^3^)	4.63 ± 0.64 (n = 75)^b^	2.28	–	–	2.02 ± 0.72 (n = 58)	1.00

^a^*p* < 0.01, as compared with the background.

^b^*p* < 0.01, compared with the non-activity period.

### Particle size and size distribution

**Figure 3:**
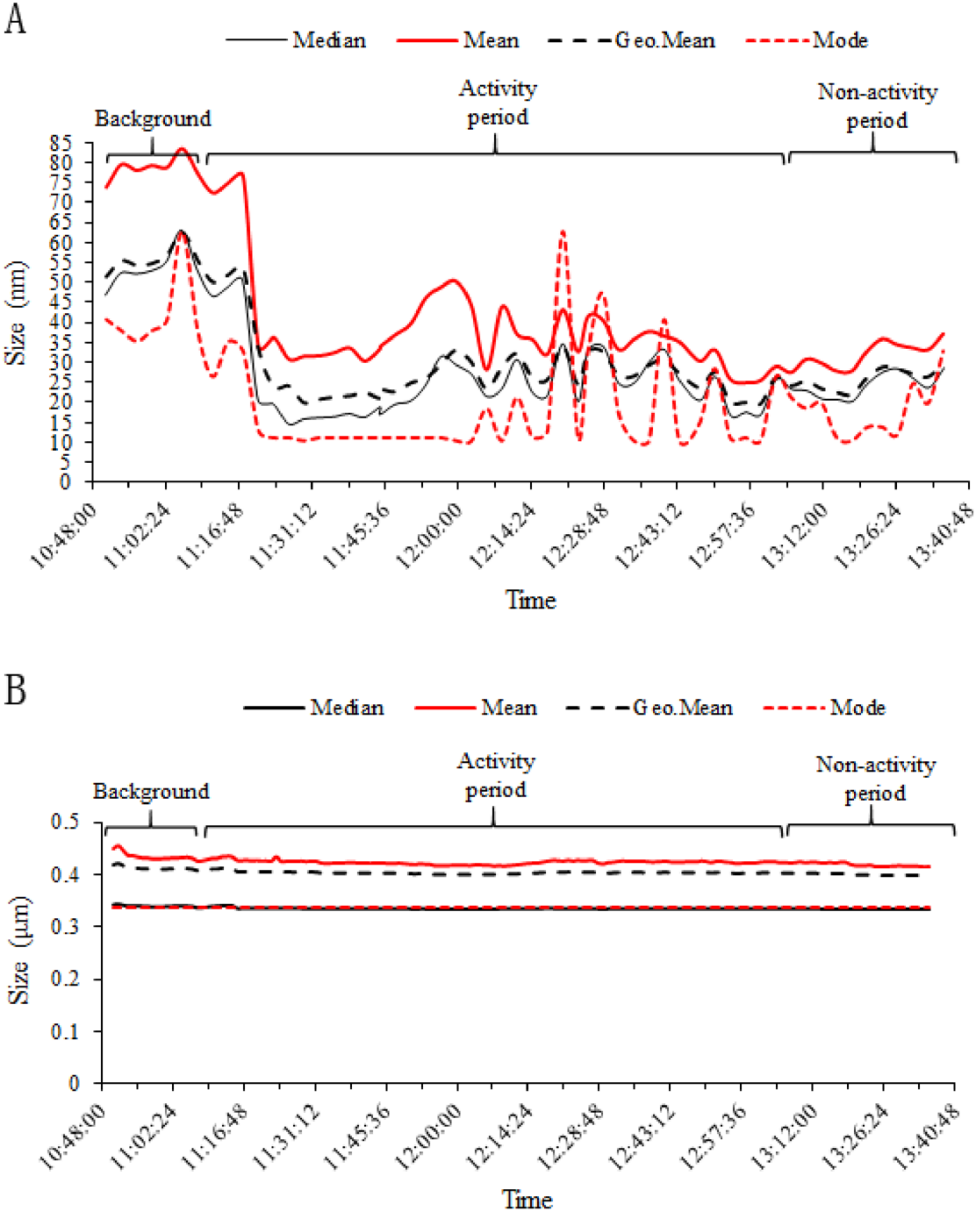
Temporal variations in mode, median, mean, and geometric mean particle sizes. (**A**) Mode, median, mean, and geometric mean size of the particles monitored by SMPS at background and operation locations. (**B**) Mode, median, mean, and geometric mean size of the particles monitored by optical particle sizer (OPS) at the background and operation locations.

Figure 3 shows the temporal variations in the mode, median, mean, and geometric sizes of the ultrafine particles from cooking and the background. The median, mean, geometric mean, and mode sizes of particles, which were determined using the SMPS, remained relatively stable during the activity period, ranging from 15 to 50 nm. In contrast, these particle sizes ranged from 45 to 85 nm before operations started. The mode size of the particles, which was determined by the SMPS, exhibited greater variations. The variations in particle size monitored by OPS were different from those determined by the SMPS. The changes in the median, mean, geometric mean, and mode sizes of particles remained steady, ranging from 0.35 to 0.45 μm. The order of particle sizes by type monitored by SMPS or OPS was as follows: mode < median < geometric mean < mean.

**Figure 4:**
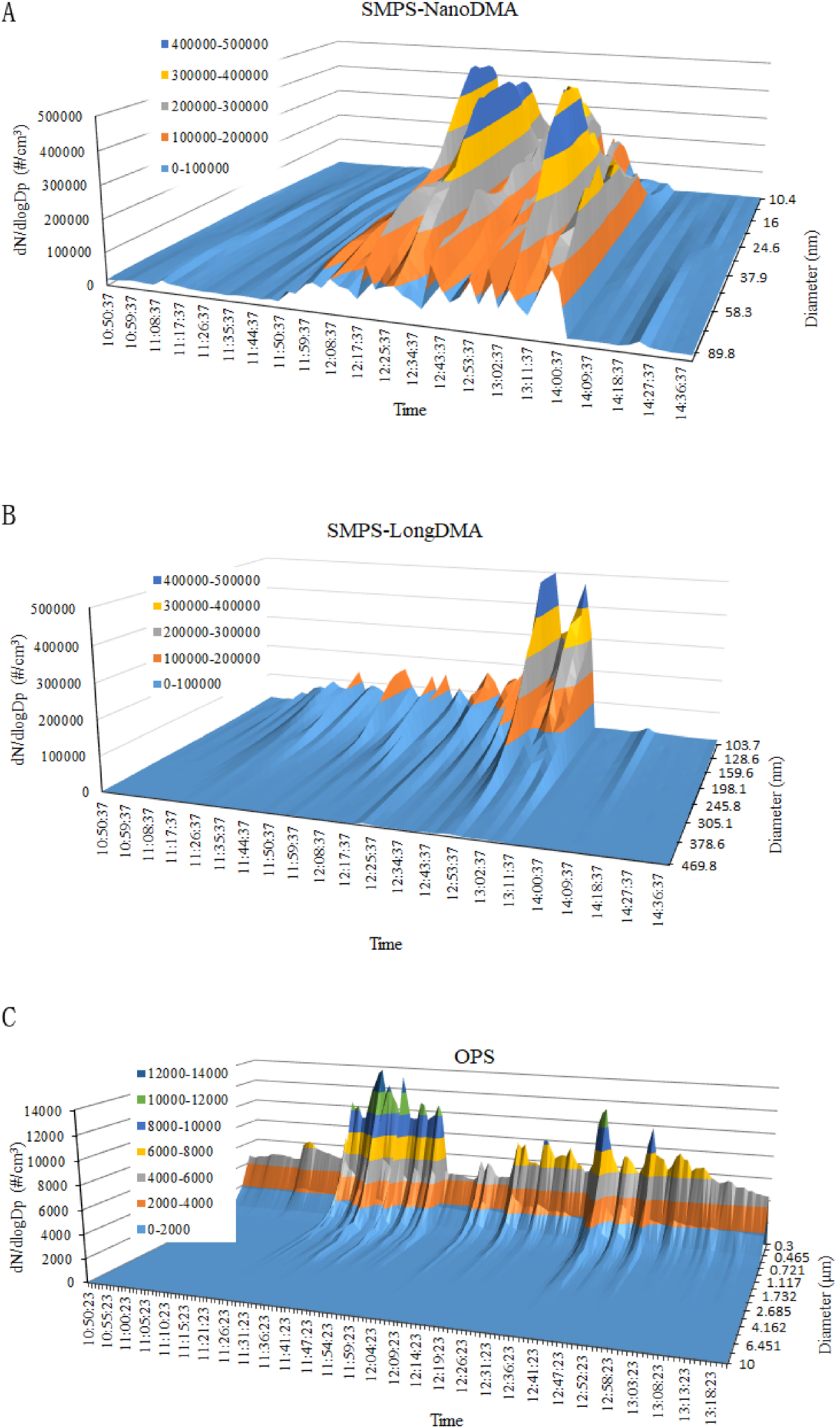
Real-time particle size spectrum. (**A**) scanning mobility particle spectrometer (SMPS) with Nano dust/aerosol monitor (DMA); (**B**) SMPS with Long DMA; (**C**) optical particle sizer (OPS). Most of the particles were smaller than 100 nm, and the highest number reached 5.5 × 10^5^ pt/cm^3^ at 10.4 nm and 100 nm.

Figure 4 shows a typical particle size distribution (dN/dLogDp, particle/cm^3^) as a function of time, as measured by SMPS and OPS. The particle diameters were 10.4–96.5 nm for the SMPS named Nano DMA, 103.7–469.8 nm for the SMPS named Long DMA, and 0.3–10 μm for OPS. The total NC of the particles with a small size increased at approximately 11:50 am around 30 min after cooking. Particles during the activity period were mainly smaller than 150 nm, and the highest number reached 5.5 × 10^5^ pt/cm^3^ (Fig. 4A), which appeared at 10.4 nm and 100 nm. The number of particles greater than 150 nm was lower than 2 × 10^5^ pt/cm^3^. The OPS measured showed that particles larger than 374 nm presented concentrations lower than 1.4 × 10^4^ pt/cm^3^. The NC of the particles, which ranged from 10.4 nm to 0.4 μm, significantly decreased after the non-activity period, as illustrated in Fig. 4.

### Risk assessment and risk management

Table 5 shows the results of the risk assessment from the CB tools and the suggested preventive measures according to the NIOSH regulation^29^. The hazard band ratios given by the Stoffenmanager Nano and Nanotool were 0.8 and 0.75, respectively. The exposure band ratios given by different CB tools were the same (0.75). The RRs given by the two tools were 0.67 and 0.75, respectively. The capture point velocity of exhaust hood is 0.2 m/s measured by the hot-wire anemometer, which is far below the regulation of technical specifications for capture velocity for LEV facilities (AQ/T4274-2016)^30^ in China. The high exposure level and risk level suggested that protective measures should be developed and implemented. According to the NIOSH regulation, five steps are needed, such as eliminating, substitution, engineering controls, administrative controls, and personal protective equipment (PPE). As eliminating and substitution of ultrafine particles are not be possible during cooking, the following hierarchy of controls should be used to implement^29^: engineering controls, administrative controls, and PPE. The current control measures and the additional control measures to be improved are listed in Table 5.

**Table 5:** The outcomes of risk assessment and management.

Tools	Hazard band ratio	Exposure band ratio	RR	Existing control measures	Additional control measures to be improved
Stoffenmanager Nano	0.8	0.75	0.67	(1) Engineering controls: the gas stove was equipped with a LEV. The capture velocity of LEV was insufficient (2) Occupational health management system: regular occupational health training, reduced exposure time, and occupational health examinations for workers. The preventative maintenance schedule and sensitive indicators for ultrafine particles were missing	(1) Engineering controls: the exhaust speed of LEV needs to be increased, including the redesign of hood for total enclosure (2) Occupational health management system: the preventative maintenance schedule for ensuring the effectiveness of engineering control measures should be established; sensitive indicators for ultrafine particles need to be developed in occupational health examination (3) PPE: the NIOSH-certified N95 or P100 filtering facepiece respirators should be used, and regular inspection should be conducted to ensure workers are properly wearing the PPE
Nanotool	0.75	0.75	0.75		

## Discussion

Considerable progress has been made in research on cooking emissions in China over the last 20 years. Studies have focused on the concentrations of PM, PAHs and VOCs, emission rates, and influential factors (such as the cooking method, types of oil, and kinds of stoves). In this study, ultrafine particles from the cooking process were collected, and their exposure characteristics and risk assessment were investigated. To our knowledge, this is the first study to comprehensively evaluate ultrafine particles' exposure risks and control measures in a Chinese restaurant using a combination of exposure characterization and the CB tools.

The total NC and total respirable MC during cooking were significantly higher than those from the background or during the non-activity period (Table 4). The CR of total NC was up to 5.4, and the CR of MC (PM_1_ and PM_2.5_) were similar (3.85 and 3.93). These results suggested that the cooking process conducted in the Chinese restaurant was able to generate nanoparticles at high levels (in particular, in higher NC and SAC). Similarly, the personal NC during cooking was significantly higher than that during the non-activity period, including the total NC, which provided direct evidence that workers were exposed to high NC levels of ultrafine particles during the cooking process. This result was supported by studies on emissions of air pollutants from cooking, which reported that the average NC of ultrafine particles ranged between 0.354 × 10^6^ and 6.643 × 10^6^ pt/cm^3^, and the MC of PM2.5 released during cooking is approximately 28.7–44,920 μg/m^3^ in commercial kitchens^31–34^. The phenomenon that personal NC was higher than the total NC suggested that the real exposure concentrations were higher than expected, while most of the monitoring was conducted on the total NC. The SAC in the alveolus and TB models during cooking was higher than those from the background or during the non-activity period (Table 4), which was consistent with the total NC. This result was confirmed by a study on the deposition of ultrafine particles in the lungs, outdoors and indoors during cooking, which found that the number of particles that had been deposited deep into the lungs during cooking was 10 times higher indoors than outdoors^35^.

As Fig. 2 shows, the temporal variations of total NC, SAC, and personal NC exhibited an activity-related characteristic, which consisted of a background level, a starting phase, a steady-state phase and finally, a decline phase. The total NC and SAC varied with working activities, while total MC was not consistent with activity in time. The correlation between total NC and SAC was 0.786, the correlation between NC and MC was 0.218, the correlation between SAC and MC was 0.380. The correlations between total NC, SAC, and MC has been explored in previous studies about ultrafine particles using real-time measurements^36,37^, which supported our finding. The high correlation between NC and SAC might be related to the fact that the 3007 counts the number of particles, the AeroTrak charges particles in a unipolar charge and then converting the number of particles into surface area. The reason for the phenomenon that MC was not increased higher than background immediately after cooking started might be that, with an average mode size of 40, such a nano-size of particles does not interact strongly with electromagnetic radiation of optical wavelength, and cannot be detected efficiently by a light-scattering photometer^38^. This result suggested that the MC was not not as sensitive as the NC or the SAC in mesuring ultrafine particles. This finding was confirmed by a field study that reported that the total NC of nanoparticles was 100 times higher than the exposure limit, and MC was no longer an appropriate evaluation metric^39^. The reason of differences in trends for temporal variations of total NC and personal NC may be related with the range of particle size meaured by different instruments, and the monitoring location. The personal NC obtained from Discmini was fixed approximately 1.3 m above the floor and 30 cm away from the breathing zone of chef, which can provide a more realistic reaction of the variation of primary released particles. While the total NC obtained from 3007 which was located farther than Discmini. The personal NC presented more sensitive to the cooking activity. As reported in a previous study, the PM level could be much higher when the oil temperature exceeded the oil smoke point^1^. This was consistent with our results, which showed that the NC of the chef got from Discmini fluctuated greatly during the activity period as the stoves fired intermittently.

The results of temporal variation in mode, median, mean, and geometric sizes showed that their trends were similar. The distance of change in arithmetic mean, medium, and geometric mean sizes was similar, but less sensitive than that for modal size. The size distribution data from SMPS was different from that obtained from OPS. One of the reason could be the different size range of instruments, as the range of particles SMPS measured was 10–487 nm, and the range of OPS measured was 0.3–10 μm. Another reason might be the different principles of detection, which were electrical mobility diameter for SMPS and light scattering equivalent diameter for OPS. As Fig. 4 showed, the predominant particles were below 150 nm and changed a lot, while the particles with size bigger than 0.3 μm occupied a small percentage. Figure 4 showed that the particles released from the cooking process were mainly smaller than 150 nm. The highest NC appeared at 10.4 nm and 100 nm, which presented a bimodal size distribution. The high NC at 100 nm suggested that the ultrafine particles in cooking fumes could agglomerate. The total NC of particles less than 100 nm was approximately 10 times higher than that of particles above 100 nm. This was confirmed by a previous study on environmental chemistry in a family kitchen, which found that cooking particles were predominantly in the ultrafine mode^40^.

The exposure band ratio, hazard band ratio, and RR of this scenario given by two CB tools were similar (higher than 0.65), which belonged to high levels. The high proportion of carcinogens in cooking fumes^41^ supported the result of high hazard levels obtained from CB tools. The high exposure risk was confirmed with the results of NC, MC, SAC, and personal NC, which showed high CRs during the cooking process (Table 4). Epidemiological studies have reported that cooking oil fumes contain many carcinogens^42^, and their exposure could increase the risk of cancer^43,44^, which provides evidence for the high hazard risk. Controlling exposures to occupational hazards is the fundamental method of protecting workers under high exposure risk. According to the NIOSH regulation, a hierarchy of controls, including elimination, substitution, engineering controls, administrative controls, and personal protective equipment, has been used as a means of determining how to implement feasible and effective controls^29^. For the restaurant investigated in this study, elimination and substitution are not viable options, the most desirable alternative for mitigating occupational hazards is to employ engineering controls. Both of outcomes of two CB tools suggested preventive measures included LEV, which suggested that the existing LEV in the restaurant was not effective. The existing LEV in the restaurant is a kind of exhaust hood with suction fans, which is 0.8 m away from gas stove. The capture point velocity of exhaust hood is only 0.2 m/s, which is far below the regulation of China and the possible reason of low capture effective. The results of concentrations and risk assessment suggested that the existing control measures do not reduce exposures to an acceptable level. The reason might be the low velocity, the location of the exhaust hood mouth, the rising airflow from a hot process. To protect workers in similar restaurants, the most desirable alternative is to employ the following additional measures before detailed regulations being enacted: (1) the exhaust speed of LEV at the gas stove needs to be increased, including the redesign of hood for total enclosure; (2) the preventative maintenance schedule for ensuring the effectiveness of engineering control measures should be established; sensitive indicators for ultrafine particles need to be developed in occupational health examination; (3) the NIOSH-certified N95 or P100 filtering facepiece respirators should be used and regular inspection should be conducted to ensure workers are properly wearing the PPE.

## Conclusions

In summary, the workers during cooking are at high risk due to exposure to high levels of ultrafine particles associated with working activity and with a bimodal size distribution. To reduce the risk, the existing control strategies, including engineering control, management control, and personal protection equipment need to be improved in this restaurant. The baseline data provided by this study can be used to develop standards for ultrafine particle exposure assessment and to design effective exposure controls in restaurants. More field investigations are needed to improve the exposure control strategies for the Chinese restaurant.
